# Predicting bee community responses to land-use changes: Effects of geographic and taxonomic biases

**DOI:** 10.1038/srep31153

**Published:** 2016-08-11

**Authors:** Adriana De Palma, Stefan Abrahamczyk, Marcelo A. Aizen, Matthias Albrecht, Yves Basset, Adam Bates, Robin J. Blake, Céline Boutin, Rob Bugter, Stuart Connop, Leopoldo Cruz-López, Saul A. Cunningham, Ben Darvill, Tim Diekötter, Silvia Dorn, Nicola Downing, Martin H. Entling, Nina Farwig, Antonio Felicioli, Steven J. Fonte, Robert Fowler, Markus Franzén, Dave Goulson, Ingo Grass, Mick E. Hanley, Stephen D. Hendrix, Farina Herrmann, Felix Herzog, Andrea Holzschuh, Birgit Jauker, Michael Kessler, M. E. Knight, Andreas Kruess, Patrick Lavelle, Violette Le Féon, Pia Lentini, Louise A. Malone, Jon Marshall, Eliana Martínez Pachón, Quinn S. McFrederick, Carolina L. Morales, Sonja Mudri-Stojnic, Guiomar Nates-Parra, Sven G. Nilsson, Erik Öckinger, Lynne Osgathorpe, Alejandro Parra-H, Carlos A. Peres, Anna S. Persson, Theodora Petanidou, Katja Poveda, Eileen F. Power, Marino Quaranta, Carolina Quintero, Romina Rader, Miriam H. Richards, T’ai Roulston, Laurent Rousseau, Jonathan P. Sadler, Ulrika Samnegård, Nancy A. Schellhorn, Christof Schüepp, Oliver Schweiger, Allan H. Smith-Pardo, Ingolf Steffan-Dewenter, Jane C. Stout, Rebecca K. Tonietto, Teja Tscharntke, Jason M. Tylianakis, Hans A. F. Verboven, Carlos H. Vergara, Jort Verhulst, Catrin Westphal, Hyung Joo Yoon, Andy Purvis

**Affiliations:** 1 Department of Life Sciences, Imperial College London, Silwood Park Campus, Buckhurst Rd, Ascot, Berkshire SL5 7PY, UK; 2 Department of Life Sciences, Natural History Museum, Cromwell Road, London SW7 5BD, UK; 3 Nees Institute for Plant Biodiversity, University of Bonn, Meckenheimer Allee 170, 53115 Bonn, Germany; 4 Laboratorio Ecotono, INIBIOMA (CONICET - Universidad Nacional del Comahue), Quintral 1250, 8400 Bariloche, Río Negro, Argentina; 5 Institute for Sustainability Sciences, Agroscope, Reckenholzstrasse 191, 8046 Zurich, Switzerland; 6 Smithsonian Tropical Research Institute, Apartado 0843-03092, Balboa, Ancon, Panama City, Republic of Panama; 7 Biosciences, Nottingham Trent University, Nottingham, NG11 8NS, UK; 8 Centre for Agri-Environmental Research, School of Agriculture, Policy and Development, University of Reading, Earley Gate, Reading, RG6 6AR, UK; 9 Science & Technology Branch, Environment and Climate Change Canada, 1125 Colonel By Drive, Carleton University, Ottawa, Ontario K1A 0H3, Canada.; 10 Alterra, Part of Wageningen University and Research, P.O. Box 47, 6700 AA WageningenI, Netherlands; 11 Sustainability Research Institute, University of East London, 4-6 University Way, Docklands, London E16 2RD, UK; 12 Grupo de Ecología y Manejo de Artrópodos, El Colegio de la Frontera Sur (ECOSUR), Carretera Antiguo Aeropuerto km 2.5. Tapachula, 30700 Chiapas, Mexico; 13 CSIRO Land and Water, Canberra, ACT 2601, Australia; 14 British Trust for Ornithology (Scotland), Biological and Environmental Sciences, University of Stirling, FK9 4LA, UK; 15 Department of Landscape Ecology, Institute for Natural Resource Conservation, Kiel University, Olshausenstrasse 75, 24118 Kiel, Germany; 16 Department of Biology, Nature Conservation, University Marburg, Marburg, Germany; 17 Institute of Integrative Biology, ETH Zurich, Switzerland; 18 Applied Entomology, ETH Zurich, Schmelzbergstr. 7/LFO, 8092 Zurich, Switzerland; 19 RSPB, Scottish Headquarters 2 Lochside View, Edinburgh Park, Edinburgh, EH12 9DH, UK; 20 Institute for Environmental Sciences, University of Koblenz-Landau, Fortstr. 7, 76829 Landau, Germany; 21 Conservation Ecology, Faculty of Biology, Philipps-Universität Marburg, Karl-von-Frisch-Str. 8, 35032 Marburg, Germany; 22 Dipartimento di Scienze Veterinarie, Viale delle Piagge 2, 56100, Pisa, Universitá di Pisa, Italia; 23 Department of Soil and Crop Sciences, Colorado State University, Fort Collins, CO 80523, USA; 24 School of Life Sciences, University of Sussex, BN19QG, UK; 25 Helmholtz Centre for Environmental Research - UFZ, Department of Community Ecology, Theodor-Lieser-Straβe 4, 06120 Halle, Germany; 26 Agroecology, Department of Crop Sciences, Georg-August-University Göttingen, D-37077 Göttingen, Germany; 27 School of Biological Sciences, Plymouth University, Plymouth PL4 8AA, UK; 28 Department of Biology, University of Iowa, Iowa, USA; 29 Agroscope, Institut for Sustainability Sciences, CH-8046 Zurich, Switzerland; 30 Department of Animal Ecology and Tropical Biology, Biocenter, University of Würzburg, Am Hubland, 97074 Würzburg, Germany; 31 Justus-Liebig University, Department of Animal Ecology, Heinrich-Buff-Ring 26-32, 35392 Giessen, Germany; 32 Institut für Systematische und Evolutionäre Botanik, Switzerland; 33 Dept. for Ecology and Conservation of Fauna and Flora, Federal Agency for Nature Conservation (Bundesamt für Naturschutz, BfN), Konstantinstrasse 110, D-53179 Bonn, Germany; 34 Institut de Recherche pour le Développement (IRD), 93143 Bondy Cedex, France; 35 Centro Internacional de Agricultura Tropical (CIAT), Tropical Soil Biology and Fertility Program, Latin American and Caribbean Region, Cali, Colombia; 36 INRA, UR 406 Abeilles et Environnement, CS 40509, F-84914 Avignon, France; 37 School of BioSciences, University of Melbourne, Parkville VIC 3010, Australia; 38 New Zealand Institute for Plant and Food Research Ltd, Private Bag 92169, Auckland Mail Centre, Auckland 1142, New Zealand; 39 Marshall Agroecology Ltd, 2 Nut Tree Cottages, Barton, Winscombe BS25 1DU, UK; 40 Departamento de Biología, Facultad de Ciencias, Universidad Nacional de Colombia, Sede Bogotá, Colombia; 41 University of California, Riverside Department of Entomology, 900 University Avenue, Riverside, CA 92521, USA; 42 Department of Biology and Ecology, Faculty of Science, University of Novi Sad, 21000 Novi Sad, Serbia; 43 Department of Biology, Lund University, SE-223 62 Lund, Sweden; 44 Swedish University of Agricultural Sciences, Department of Ecology, Box 7044, SE-750 07 Uppsala, Sweden; 45 RSPB, UK Headquarters The Lodge, Sandy, Bedfordshire, UK; 46 Laboratorio de Investigaciones en Abejas, LABUN, Departamento de Biología, Facultad de Ciencias, Universidad Nacional de Colombia, Carrera 45 No. 26-85, Edif. Uriel Gutiérrez, Bogotá D.C., Colombia; 47 Corporación para la Gestión de Servicios Ecosistémicos, Polinización y Abejas - SEPyA, Bogotá D.C., Colombia; 48 School of Environmental Sciences, University of East Anglia, Norwich NR47TJ, UK; 49 Laboratory of Biogeography & Ecology, Department of Geography, University of the Aegean, 81100 Mytilene, Greece; 50 Entomology Department, Cornell University, Ithaca, NY 14850, USA; 51 Botany, School of Natural Sciences, Trinity College Dublin, Dublin 2, Ireland; 52 CREA-ABP, Consiglio per la ricerca in agricoltura e l’analisi dell’economia agraria, Centro di ricerca per l’agrobiologia e la pedologia, Via di Lanciola 12/A, I-50125 - Cascine del Riccio, Firenze, Italy; 53 School of Environmental and Rural Science, University of New England, Armidale, New South Wales, Australia; 54 Department of Biological Sciences, Brock University, St. Catharines, Ontario, L2S 3A1, Canada; 55 Department of Environmental Sciences, University of Virginia, Charlottesville, Virginia 22904-4123, USA; 56 Blandy Experimental Farm, 400 Blandy Farm Lane, Boyce, Virginia 22620, USA; 57 Département des Sciences Biologiques, Université du Québec à Montreál, C.P. 8888, succursale Centre-ville, Montreál, Québec H3C 3P8, Canada; 58 GEES (School of Geography, Earth and Environmental Sciences, University of Birmingham, Birmingham B15 2TT, UK; 59 Department of Ecology, Environment and Plant Sciences, Stockholm University, SE-106 91 Stockholm, Sweden; 60 CSIRO, Dutton Park, QLD 4102, Australia; 61 University of Bern, Institute of Ecology and Evolution, Community Ecology, Baltzerstrasse 6, 3012 Bern, Switzerland; 62 Animal and Plant Health Inspection Service, Plant Protection and Quarantine, United States Department of Agriculture (USDA), South San Francisco, CA 94080, USA; 63 Faculty of Sciences, National University of Colombia, Medellín (UNALMED), Columbia; 64 Plant Biology and Conservation, Northwestern University, 2205 Tech Drive, O.T. Hogan Hall Rm 2-1444, Evanston, IL 60208, USA; 65 Chicago Botanic Garden, 1000 Lake Cook Rd, Glencoe, IL 60011, USA; 66 Department of Biology, Saint Louis University, 3507 Laclede Avenue, Macelwane Hall, St. Louis, MO 63103-2010, USA; 67 Centre for Integrative Ecology, School of Biological Sciences, University of Canterbury, Private Bag 4800, Christchurch 8140, New Zealand; 68 Division Forest, Nature, and Landscape, Department of Earth & Environmental Sciences, KU Leuven, Celestijnenlaan 200E, B-3001 Leuven, Belgium; 69 Departamento de Ciencias Químico-Biológicas, Universidad de las Américas Puebla, Mexico; 70 Spotvogellaan 68, 2566 PN, Den Haag, The Netherlands; 71 Department of Agricultural Biology, National Institute of Agricultural Science, RDA, Wanju-gun, Jellabuk-do, 55365, Korea

## Abstract

Land-use change and intensification threaten bee populations worldwide, imperilling pollination services. Global models are needed to better characterise, project, and mitigate bees' responses to these human impacts. The available data are, however, geographically and taxonomically unrepresentative; most data are from North America and Western Europe, overrepresenting bumblebees and raising concerns that model results may not be generalizable to other regions and taxa. To assess whether the geographic and taxonomic biases of data could undermine effectiveness of models for conservation policy, we have collated from the published literature a global dataset of bee diversity at sites facing land-use change and intensification, and assess whether bee responses to these pressures vary across 11 regions (Western, Northern, Eastern and Southern Europe; North, Central and South America; Australia and New Zealand; South East Asia; Middle and Southern Africa) and between bumblebees and other bees. Our analyses highlight strong regionally-based responses of total abundance, species richness and Simpson's diversity to land use, caused by variation in the sensitivity of species and potentially in the nature of threats. These results suggest that global extrapolation of models based on geographically and taxonomically restricted data may underestimate the true uncertainty, increasing the risk of ecological surprises.

Bees are one of the most important groups of pollinators of economic crops^1^,^2^,^3^, with both larvae and adults relying on floral products such as pollen and nectar^3^. Human impacts can reduce the diversity of pollinator assemblages^4^,^5^ and therefore can impact pollination efficiency and provision. This is a particular concern in agricultural settings, as over 35% of the volume of human food crops produced globally depend upon animal pollination to some extent^6^. Pollinator shortages can lead to reduced crop quality and yield^7^,^8^, with potentially large economic impacts^9^. There has therefore been much research into responses of bee communities to human impacts such as land-use change and intensification.

A number of syntheses have attempted to identify general trends in the response of bees to human impacts^5^,^10^. However, their datasets have often been geographically limited, with the majority of data arising from North America and Western Europe^11^. The geographic patterns of bee decline and diversity are not understood sufficiently well to ensure that such generalisations are valid^11^,^12^. If species’ responses to disturbance vary among regions, geographically-restricted models will be inadequate to support broad conclusions. The consequences of basing management strategies on extrapolations from such models could be severe, as many under-studied regions have a high economic dependency upon animal-pollinated crops^11^,^13^ and may generally have limited governmental capacity to adapt to environmental changes^14^.

Geographic variation in bee community responses could arise because differences in land-use history and practices mean that the threats facing assemblages differ across regions. Species subject to very recent disturbance may be more vulnerable, whereas extinction filters^15^,^16^,^17^ may have already removed many susceptible species from landscapes where the intensification of farming started already decades ago, such as in temperate European agricultural landscapes. Extinction debt may make matters worse still, if the full impact of land-use changes is not yet evident^18^,^19^. In addition, differences in landscape context across regions can influence species’ responses. For instance, Winfree *et al*.^5^ found that habitat loss and fragmentation significantly affected bee communities, but only in areas where little natural habitat still remained.

Bee community responses may also vary regionally because community composition varies geographically. Taxa can differ in their intrinsic susceptibility to land-use change and intensification, through having different functional response traits^20^,^21^,^22^, the distribution of which within a community can affect resilience to pressures^23^. A geographic bias towards North America and Western Europe has also resulted in a taxonomic bias; for instance, bumblebees (Apidae: *Bombus*) are particularly diverse in these areas, whereas large areas of the world have no native bumblebee species (e.g., most of Africa and Australasia). In addition, bumblebees are large, often abundant species with long flight seasons and relatively slow flight, making them fairly easy to sample and, in many cases, to identify. Bumblebees may be more or less sensitive than other bees due to their ecological traits and habitat requirements^24^, which have been shown to influence responses to human impacts and vulnerability to decline^25^,^26^. In addition, bumblebees have shown clearer declines than other bees in North America^25^ and some European countries^27^, so they may be atypical of broader bee diversity.

We compiled a global dataset of bee diversity from published sources of bee assemblages in sites differing in pressures such as land use, and used this to explore whether models of responses to human impacts are robust against geographic and taxonomic biases. Specifically, we hypothesized that bee responses to land-use pressures should vary significantly with region and with taxonomic group (i.e., bumblebees or other bees) and so models and projections will not be transferable across regions and taxa. Improved understanding in this area will help to clarify whether knowledge based on a few regions and taxa is sufficient to underpin policy decisions as well as highlight systems for future study.

## Methods

### Data Collation

Data were sought from the literature where bee species abundance and/or occurrence were reported for multiple sites. Suitable papers were identified by searching Web of Science at various times from 2011 to 2015, as well as searching journal alerts and assessing references cited in reviews. Papers were further considered if more than one site was sampled for bee diversity using the same sampling method in the same season and geographic coordinates of each site were available. Papers were prioritised if their data were collected from February 2000 onwards, so that biodiversity data could be matched with remote-sensed data from NASA’s Moderate Resolution Imaging Spectroradiometer (MODIS). Data were supplemented with sources found through the PREDICTS project (www.predicts.org.uk), which aims to develop global statistical models of how local biodiversity responds to human impacts^28^. The database presented here is not a comprehensive compilation of published sources on occurrence and abundance of bee species across sites differing in land use or intensity, because of regional differences in the ability to retrieve information about potential sources and because most researchers we contacted did not make their data available. The dataset will, however, still be useful for researchers wishing to study land-use impacts on this important taxonomic group.

Where possible we extracted site-level records of bee species (Hymenoptera: Apoidea) occurrence and abundance from suitable papers, along with data for other taxonomic groups if available. Raw data were usually not included within the papers or supplementary files, so the papers’ corresponding authors were asked for these data. Relevant data were available from 69 papers, hereafter referred to as ‘sources’ (Table 1). Each source contains one or more studies, where a study is defined as the set of samples within the same country that were taken using the same methodology. By defining studies in this way, we reduce the impact of broad-scale biogeographic differences in diversity and avoid the confounding effects of methodological differences: within, but not between, studies, diversity data can be compared among sites in a straightforward fashion. Differences in sampling effort within a study were corrected for when necessary by dividing abundance by the sampling effort unit. This assumes a linear relationship between abundance and sampling effort; generalised additive models suggested that this assumption was appropriate (gamm4 package^29^, see Supplementary Data S1 for details). Within each study, we recorded any blocked or split-plot design. The major land-use class and use intensity at each site were assessed based on information in the associated paper, using the scheme described in Hudson *et al*.^28^ (reproduced in Supplementary Table S1). Briefly, land use was classified as primary vegetation (native vegetation not known to have ever been completely destroyed), secondary vegetation (where the primary vegetation has been completely destroyed; this can include naturally recovering, actively restored, or semi-natural sites), cropland (planted with herbaceous crops), plantation forest (planted with crop trees or shrubs), pasture (regularly or permanently grazed by livestock) or urban (areas with human habitation, where vegetation is predominantly managed for civic or personal amenity). Use intensity was classified according to a three point scale: low, medium and high intensity. For instance, high-intensity cropland would be monocultures with many signs of intensification such as large fields with high levels of external inputs, irrigation and mechanisation; medium intensity cropland would only show some, but not all, features of higher intensity cropland; low-intensity would refer to small fields with mixed crops and little to no external inputs, irrigation or mechanisation. In one data source, information on the use intensity was unavailable at the site-level, so information at the landscape level was used.

The dataset contained 111 studies from 69 sources and 3211 within-study sites (Table 1). This amounted to 195,357 species diversity measurements (i.e., bee taxa and other taxa, Table 1), including 107,176 measurements of bee diversity (a single measurement being, for example, the abundance of a given species at a given site; see Supplementary Data S2 for species list).

### Analysis

For this analysis, we did not include studies that recorded only particular target species (for instance, studies that were only interested in the abundance of a single species across sites), so that site-level diversity measures would be meaningful. The final dataset for the analysis included 101,524 diversity records from 837 bee species at 2421 sites from across the globe (North America: 239 sites; Central America: 103; South America: 176; Western Europe: 1211; Northern Europe: 325; Eastern Europe: 64; Southern Europe: 50; Middle and Southern Africa: 39; South Eastern Asia: 31; Australia and New Zealand: 183). In this reduced dataset, many combinations of land use and use intensity had too few sites to permit robust modelling. The data were therefore aggregated to give a variable of combined Land Use and Intensity (LUI) with the following levels: primary vegetation, secondary vegetation, low-intensity cropland, medium-intensity cropland, high-intensity cropland, pasture, plantation forest and urban. All LUI levels had at least 170 sites, except for plantation forest and urban areas, which were scarce in the dataset with only 105 and 94 sites respectively. Sites were also classified by region and subregion (according to United Nations classifications), with Middle and Southern Africa combined into a single category to increase the sample size.

For each site, we calculated three measures of bee community diversity as our response variables: total abundance, within-sample species richness and Simpson’s diversity. Simpson’s diversity was calculated as:


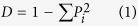


where *P*_*i*_ is the proportion of individuals belonging to species *i*. We use Simpson’s diversity as it stabilises faster than species richness and other diversity measures as specimens accumulate^30^.

As total abundance measurements are not necessarily integers (e.g. densities and effort-corrected measures), use of the Poisson error structure was not possible, so total abundance was ln + 1 transformed before modelling to normalise residuals and equalise variance. Total abundance and Simpson’s diversity were modelled using Gaussian errors (model-checking showed that these treatments were appropriate). Species richness was modelled with Poisson error distribution and log-link function; there was evidence of significant overdispersion in these models so an observation-level random effect was included to account for this (i.e., a Poisson-lognormal model)^31^.

All analyses were carried out using R 3.1.0^32^. We constructed models for each response variable, using mixed-effects models (lme4 package^33^) to account for non-independence of data due to differences in collectors (‘source’), sampling methodologies and biogeographic source pools (‘study’) and the spatial structure of sites (‘block’); the initial random-effects structure was therefore block nested within study within source. The initial fixed-effects structure of models included LUI, subregion and their interaction. Subregion is treated as a fixed rather than random effect as we are interested in testing the effect, rather than simply estimating the variance associated with geographic subregion. We test differences in responses to LUI among subregions rather than assessing how responses vary with the latitude and longitude of sites, as subregions represent political differences in land-use patterns and data availability, as well as to some extent reflecting biogeographical differences in community composition.

The best random-effects structure was assessed using likelihood ratio tests^34^, with models fit using Restricted Maximum Likelihood for total abundance and Simpson’s diversity, and Maximum Likelihood for species richness. We then attempted to simplify the fixed-effects structure using backwards stepwise model simplification and likelihood ratio tests, with models fit using Maximum Likelihood^34^,^35^,^36^. Significance of terms in the minimum adequate models were assessed using Type II Wald Chi Square Tests^37^. However, to better appreciate the uncertainty in the models^38^, if the interaction between LUI and subregion remained in the minimum adequate model, we also constructed the following models: additive model (with LUI and subregion included as additive effects); LUI only (univariate model); and subregion only (univariate). We then compared the explanatory power and predictive error of the interactive model with these simpler alternatives.

Explanatory power was calculated using the MuMIn package in R^39^, as the marginal and conditional R^2^_glmm_ values: i.e., the variance explained by fixed effects alone and by fixed and random effects combined, respectively^40^. Predictive error was calculated as the Mean Squared Error (MSE) from ten-fold cross validation, where the model was iteratively fit to nine-tenths of the data (training set), and validated on the final tenth (validation set); we did this by randomly assigning sites into ten approximately equal-sized groups^41^. As the data are structured, the training data may not be fully independent of the validation data^42^, but any bias in prediction error that this causes will apply equally to all models being compared as the random effect structures are identical. In addition, some combinations of explanatory variables only occur in few studies or sources; splitting the dataset by these higher-level strata would mean that some combinations would rarely appear in the training data, leading to overestimates of predictive error. MSE was decomposed into measures of bias and variance, which give an indication of the accuracy and precision of predictions respectively^43^ (See Supplementary Methods for details).

The dataset was then subset to include only studies where both bumblebees (Apidae: *Bombus*) and other bees were sampled (bumblebees contributed over 19% of the bee abundance records); this resulted in 1636 sites from 47 studies. We calculated the site-level diversity measures separately for each group and fitted the initial model with a three-way interaction between LUI, subregion and taxonomic group (*Bombus* or otherwise). The initial random structure was as above, but included a site-level random effect to account for multiple samples (bumblebees and other bees) being taken from the same site. As above, we first attempted to simplify the initial model (both in terms of random effects and then fixed effects) and, if the initial three-way interaction remained in the model, compared the explanatory power and predictive error with simpler models, where responses to LUI were permitted to vary with subregion (LUI, subregion and their interaction) or with taxonomic group (LUI, taxonomic group and their interaction).

To further understand heterogeneity in community response to LUI, planned comparisons were performed (multcomp package^44^). Within each subregion (and each taxonomic group, if assessed), we tested for differences between natural vegetation (primary vegetation) and all other land uses; between semi-natural vegetation (secondary vegetation) and all other land uses (except primary); whether low-intensity cropland differed from medium-intensity cropland; and whether medium-intensity cropland differed from high-intensity cropland. To avoid rank-deficiency, LUI and subregion were collapsed into a single factor in these models. Not all comparisons were possible in all subregions. Multiple comparisons were corrected for using the False Discovery Rate method to adjust significance values^45^,^46^.

An alpha value of 0.05 was used in all tests for significance. Spatial autocorrelation was assessed in residuals of minimum adequate models using Moran’s I, for each study in turn (spdep package^47^,^48^). As multiple tests are carried out, we expect 5% of these to be significant by chance so we additionally test whether the proportion of studies showing autocorrelation exceeds this expected proportion (using a one-sided Chi squared test).

## Results

**Figure 1:**
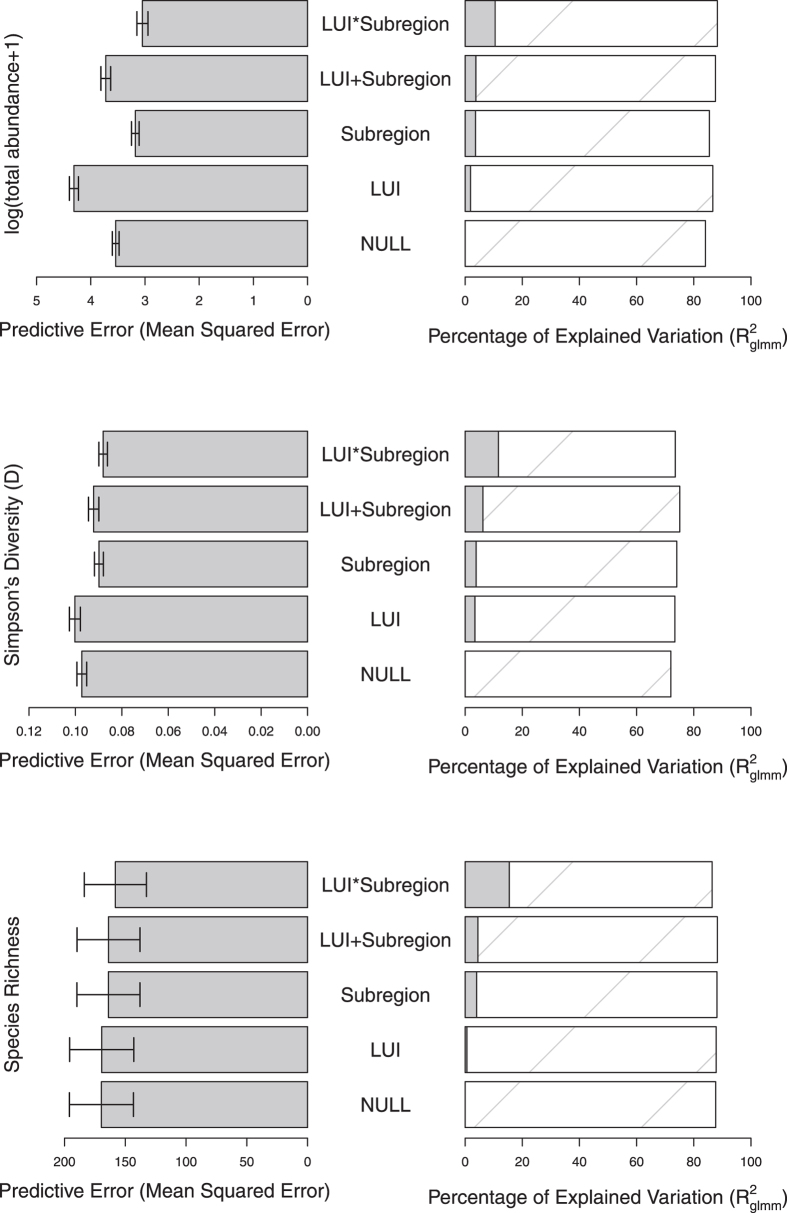
The predictive error and explanatory power of models that include only the intercept (NULL), LUI alone, subregion alone, additive effects, or interactive effects. LUI = Land Use and Intensity. For explanatory power, solid bars show the marginal R^2^_glmm_ (the variance explained by fixed effects) and the hashed bars show the conditional R^2^_glmm_ (the variance explained by both random and fixed effects). Error bars show the standard error of the mean predictive error across 10 folds of cross validation. Note that the predictive error should only be compared among models assessing the same response variable, as absolute values depend on the measurement scale.

For total abundance, Simpson’s diversity and species richness, the minimum adequate models were those in which responses to LUI were free to vary among geographic subregions. These models also always had the greatest explanatory power and were always among the models having the lowest predictive error (Fig. 1). Overall, however, explanatory power of fixed effects alone was fairly low, with most variation instead being attributed to random effects, which is not surprising given the variation in sampling methodology and effort among studies and sources.

**Figure 2:**
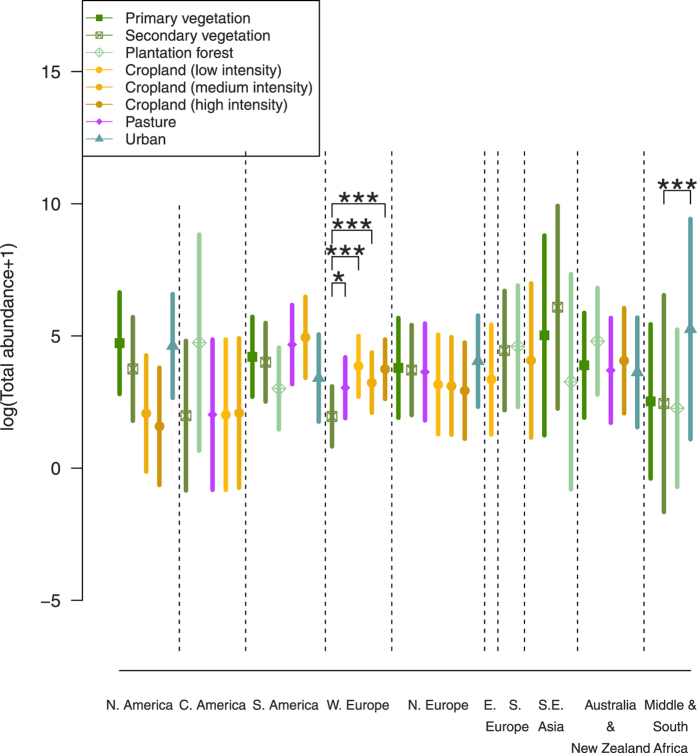
Predicted means of total (logged) abundance of bees for different land-use classes in each subregion, with 95% confidence intervals. Also shown are significant results of multiple comparisons, testing differences between natural (Primary vegetation) and semi-natural land uses (Secondary vegetation) to human-dominated land uses, and differences between low, medium and high intensity cropland (*p < 0.05, **p < 0.01, *** *p* < 0.001).

For total abundance, the interaction between LUI and subregion explained a highly significant amount of variation (*χ*^2^ = 133.15, df = 27, *p* < 0.0001) (Fig. 2), which resulted in this model having the lowest predictive error compared to simpler models. The interaction between LUI and subregion was also significant for Simpson’s diversity (*χ*^2^ = 66.48, df = 27, *p* < 0.0001) and species richness (*χ*^2^ = 96.41, df = 27, *p* < 0.05), although the predictive error was not much lower than for models based on subregion alone. In all cases, the interactive model had slightly higher bias than some simpler models, but the lowest variance (See Supplementary Table S2).

As Fig. 2 shows, the response of total bee abundance to land use differs significantly among regions. In Western Europe, agricultural land maintained higher bee abundances than secondary vegetation (low-intensity cropland: z = 8.21; medium-intensity cropland: z = 5.33; high-intensity cropland: z = 9.19; all *p* < 0.0001; pasture: z = 4.18, *p* = 0.012). Low-intensity cropland also maintained higher diversity than secondary vegetation (Simpson’s diversity: z = 4.22, *p* = 0.017) and medium-intensity cropland (Simpson’s diversity: z = −5.68, *p* < 0.0001; Species richness: z = −4.82, *p* = 0.0015).

In South America, bees were more sensitive to agricultural land uses: medium-intensity cropland maintained significantly lower Simpson’s diversity than secondary vegetation (z = −5.15, p = 0.00029). Urbanization had differing effects between subregions, with increased species richness (z = 5.29, *p* = 0.00022) in Middle and Southern Africa, but no strong effect detected elsewhere.

**Figure 3:**
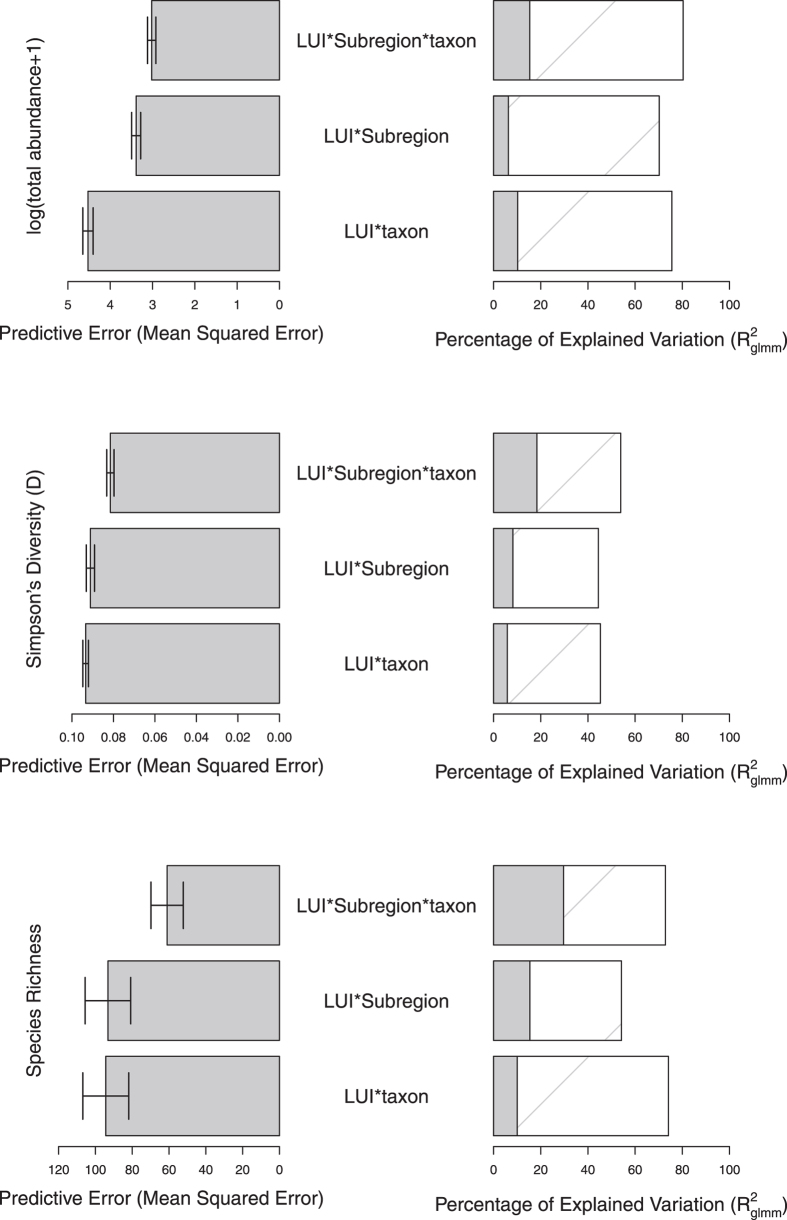
The predictive error and explanatory power of models that include three way interactions between LUI, subregion and taxon (*Bombus* or not), and models with two way interactions between LUI and taxa, or LUI and Subregion. LUI = Land Use and Intensity. For explanatory power, solid bars show the marginal R^2^_glmm_ (the variance explained by fixed effects) and the hashed bars show the conditional R^2^_glmm_ (the variance explained by both random and fixed effects). Error bars show the standard error of the mean predictive error across 10 folds of cross validation. Note that the predictive error should only be compared among models assessing the same response variable, as absolute values depend on the measurement scale.

When the dataset was split by taxon (*Bombus* vs. others), the best models for each response variable according to likelihood ratio tests included significant three way interactions between LUI, subregion and taxon (total abundance: *χ*^2^ = 217.9, df = 13, *p* < 0.0001; Simpson’s diversity: *χ*^2^ = 27.62, df = 13, *p* = 0.0102; species richness: *χ*^2^ = 76.08, df = 13, *p* < 0.0001). These models also had the greatest explanatory power and lowest predictive error, compared with simpler models (Fig. 3); for total abundance, the lower predictive error was driven by both lower bias and variance, while for Simpson’s diversity and species richness, the slightly higher bias was offset by lower variation (See See Supplementary Table S2).

Planned comparisons provided more detail into these context- and taxon-specific differences in response to human impacts. In Western Europe, bumblebees and other species respond similarly in terms of total abundance, which was higher in cropland than in secondary vegetation (e.g. in low-intensity cropland, *Bombus*: z = 6.85, *p* < 0.0001; other species: z = 9.33, *p* < 0.0001). However, bumblebee species richness tended to be higher in low-intensity cropland than secondary vegetation (z = 4.68, *p* = 0.004), which was not true for other bees (z = 1.96, n.s.). Increasing agricultural intensity also resulted in a decline in species richness, but the response was consistent across taxa: low-intensity cropland maintained higher richness than medium-intensity cropland both in bumblebees (z = −4.30, *p* = 0.016) and other bees (z = −3.75, *p* = 0.042).

Bumblebees also responded differently from other bee species in North America. *Bombus* species richness was lower in secondary vegetation relative to primary vegetation (z = −3.93, *p* = 0.027), but this was not true for other bees (z = 0.86, n.s.); similarly, total abundance was slightly lower for bumblebees (z = 3.64, *p* = 0.064) but not other species (z = 0.45, n.s.). However, other genera in North America appeared to be relatively more sensitive to medium-intensity cropland, with reduced species richness relative to both primary vegetation (z = −3.90, *p* = 0.027) and secondary vegetation (z = −3.33, *p* = 0.017). Other genera were also more sensitive to urban areas, with reduced species richness in urban sites relative to primary vegetation (z = −3.99, *p* = 0.027), while bumblebees showed no significant response (z = −2.5, n.s.).

The number of studies showing significant autocorrelation was not significantly higher than the 5% expected by chance (See See Supplementary Table S3 for details).

## Discussion

Bees are facing declines across the globe as a result of changing and intensifying land use^4^,^17^,^49^. Detailed statistical models that relate bee diversity to drivers of change have the potential to inform mitigation and conservation efforts and to help safeguard food security. However, the transferability of models based on restricted data to other regions and taxa is not guaranteed^11^,^50^. If responses to threats are context dependent, extrapolation from well known study systems could carry significant risks for biodiversity and food security. The areas where food production is most highly dependent upon animal pollination are also those for which the fewest data are available^11^,^13^,^51^, due to a lack of infrastructure and funding in many areas of the world^11^. These same areas are often poorly buffered against the disruption of ecosystem service provision from whatever cause, meaning that effects of any ecological surprises on human well-being could be more severe here than elsewhere. We have shown that bee community responses to land-use change and agricultural intensification can indeed be highly context-dependent, but whether this impacts the transferability of models depends on the facet of diversity that is of interest.

The response of total abundance, Simpson’s diversity and species richness of bee communities to land use and intensity (LUI) varied significantly with geographic region, in line with our hypotheses and with previous work in tropical regions^52^. For all response variables, the greatest predictive ability could only be achieved by allowing regional variation in responses to LUI; at the very least, it was necessary to allow regional variation in baseline diversity. This suggests that conclusions based on geographically restricted data cannot reliably be generalized to other regions. Indeed, bee community responses to agricultural intensification varied between regions; only in Western Europe was there an evident decline in diversity with increasing use-intensity of cropland, in line with previous suggestions that agricultural land-use intensity is more important in temperate than in tropical or subtropical systems^10^. The most negative impact of agriculture, however, was seen in South America, where Simpson’s diversity was significantly lower than in secondary vegetation; this is congruent with a previous meta-analysis by Gibson *et al*.^52^ that focussed on tropical areas. The effects of urbanisation likewise depended on the subregion—with increased abundance in Africa but few effects seen elsewhere—but these inferences were based on relatively few data. More data from more regions are needed to better understand the impact of urbanisation on bee communities and associated ecosystem services^53^.

It is likely that the geographic variation in responses is in part due to differences in community composition^11^, as we found that taxonomic biases towards bumblebees, which frequently dominate datasets geographically limited to North America and Western Europe, can mask the responses of other species. In Western Europe, for instance, bumblebees had higher species diversity in low-intensity cropland than in secondary vegetation, while other bee species did not show the same effect. Bumblebees have longer flight distances than many smaller bees, so may be better able to persist in more human-dominated land-uses, where foraging resources tend to be further from nesting sites^22^, and can benefit from mass-flowering crops such as oilseed rape^54^.

The effect of taxonomic group on responses to LUI also differed between subregions, suggesting that other factors may also affect generalities. For instance, geographic variation in the nature of threats may be important. Although our land-use intensity classification is applied in an equivalent fashion across regions, it remains extremely coarse. For example, high-intensity cropland may be more intensive in Western Europe than in South America, with regards to some pressures (e.g., pesticide load^55^) but potentially not others (e.g. spatial extent of monocultures). Such variation in agricultural intensification among regions (even within the same land-use intensity class) could in part be driving observed regional differences in biodiversity responses. More detailed data on different aspects of land-use intensity, such as pesticide load and fertilizer application rates, as well as data on the landscape structure, would enable a more robust and precise analysis of how responses vary across regions. This limitation still highlights, however, that models mostly underpinned by data from regions with a long and intensive history of cultivation are unlikely to provide meaningful inferences for many other regions of the world.

Variation in response among regions could also be driven by differences in community composition and therefore in the distribution of traits that may confer resistance or resilience to human impacts. Previous work has shown that trait-based models of species distributions are only transferable—even within a subregion—when land cover is similar^56^. Transferability *across* subregions is likely to be even more difficult: variability in the sensitivity of bee communities will be influenced by a complex interaction between the trait distribution (and phylogeny) across communities and variation in the threats they face. For instance, in a global analysis, species that reproduce socially were more vulnerable to isolation and pesticide use than solitary species, but were less sensitive to tillage and agricultural intensity than solitary species^20^; however, bees that reproduce solitarily are more common in temperate areas than the tropics^57^, while the distribution of these pressures also vary regionally^55^. While we only assessed how a single aspect of community composition may influence results (bumblebees vs other bees), further work into phylogenetic patterns of sensitivity may help to disentangle these two mechanisms that may be driving regional variation in responses. Another important extension to our work would be to explore the interaction of multiple threatening processes, as the pressures faced by bee communities can vary regionally^58^. For instance, competition with introduced species and fragmentation are likely to be more important drivers of native bee diversity in the Neotropics than in temperate regions^59^. While it was not the focus of this work, a spatially-explicit analysis of latitudinal gradients in vulnerability to land-use pressures may be an interesting avenue for further research, potentially highlighting other factors of the environment or community structure that could contribute to geographic variation in sensitivity. For example, species richness of bees peaks at approximately 35° latitude, in dry, Mediterranean climates^60^, rather than in the tropics (as is the case for many other groups^61^) and this variation in baseline diversity may alter both actual and detected responses to human impacts.

Although our dataset includes over 2000 sites from five continents, it is not a comprehensive compilation of published sources and is still both geographically and taxonomically biased. Africa and Asia in particular are still poorly represented and as a consequence we may still be underestimating the uncertainty in bee responses to land use in these regions. Even biomes that have high bee diversity are underrepresented; for example, only six studies were in the Mediterranean biome although bee species richness tends to peak at this latitude^60^. In addition, the explanatory power of fixed effects was fairly low, as most variation in diversity is explained by methodological differences between studies and sources in most models. Nonetheless, our analysis has important implications for pollinator research and conservation action. We show that results based on geographically and taxonomically restricted datasets may not be transferable to other regions. Responses vary across regions due to a combination of differences in the inherent vulnerability of species and variation in the nature of threats. The provision of pollination services can be influenced by the abundance^62^,^63^, species diversity^64^ and species richness^64^,^65^,^66^ of bee communities, although the relative importance of each facet of diversity appears to vary with study system^62^,^64^. Therefore, if we are to safeguard pollinators and the services they provide, research effort to enhance the representativeness (if not the amount) of available data will be needed to make context-dependent recommendations and to better understand the state of pollination services worldwide.

## Additional Information

**How to cite this article**: De Palma, A. *et al*. Predicting bee community responses to land-use changes: Effects of geographic and taxonomic biases. *Sci. Rep.* **6**, 31153; doi: 10.1038/srep31153 (2016).

## Supplementary Material

Supplementary Information

## Figures and Tables

**Table 1:** Data sources and sample sizes.

Reference	Country	Sampling years	Studies	Within-study sites	Bee taxa (% binomial)	Other taxa	mMLE
	**Afrotropic**		**3**	**39**	**77**	**2304**	
Basset *et al*.^67^^+†^	Gabon	2001–2002	1	12	51 (19.61%)	1806	70
Gaigher & Samways^68^^+†^	South Africa	2006	1	10	6 (0%)	383	nr
Grass *et al*.^69^^+†‡^	South Africa	2011	1	17	21 (9.52%)	115	100
	**Australasia**		**8**	**200**	**135**	**497**	
Blanche *et al*.^70^^+†^	Australia	2005	2	11	8 (89.36%)	17	nr
Cunningham *et al*.^71^^+†^	Australia	2007–2008	1	24	69 (100%)	0	nr
Lentini *et al*.^72^^+†^	Australia	2009–2010	1	104	36 (100%)	0	nr
Kessler *et al*.^73^^+†^	Indonesia	2004–2005	1	15	9 (0%)	24	nr
Malone *et al*.^74^^†‡^	New Zealand	2006–2007	1	2	9 (100%)	0	nr
Todd *et al*.^75^^+†^	New Zealand	2007–2008	1	20	9 (100%)	442	27.3
Rader *et al*.^21^^+†^	New Zealand	2008–2009	1	24	5 (100%)	20	nr
	**Indo-Malay**		**4**	**16**	**1**	**0**	
Liow *et al*.^76^^+†‡^	Singapore, Malaysia	1999	4	16	1 (0%)	0	3000
	**Nearctic**		**16**	**399**	**242**	**117**	
Boutin *et al*.^77^^+†^	Canada	2000	3	60	3 (0%)	116	nr
Richards *et al*.^78^^+†^	Canada	2003	3	18	127 (95.04%)	0	nr
Hatfield & Lebuhn^79^^†^	United States	2002–2003	1	120	13 (100%)	0	nr
McFrederick & LeBuhn^80^^†‡^	United States	2003–2004	2	40	5 (100%)	0	nr
Shuler *et al*.^81^^+†^	United States	2003	1	25	5 (60%)	0	nr
Winfree *et al*.^82^^+†^	United States	2003	2	80	1 (0%)	0	nr
Kwaiser & Hendrix^83^^+^	United States	2004	2	18	53 (97.22%)	1	nr
Julier & Roulston^84^^+†^	United States	2006	1	20	3 (100%)	0	250
Tonietto *et al*.^85^^+†^	United States	2006	1	18	67 (89.55%)	0	nr
	**Neotropic**		**16**	**286**	**436**	**775**	
Vázquez & Simberloff^86^^+^	Argentina	1999, 2001	1	8	25 (52%)	104	nr
Quintero *et al*.^87^^†^	Argentina	2000–2001	1	4	14 (35.71%)	38	1280
Schüepp *et al*.^88^^+†^	Belize	2009–2010	1	15	43 (100%)	65	nr
Tonhasca *et al*.^89^^+†‡^	Brazil	1997, 1999	1	9	21 (100%)	0	10
Barlow *et al*.^90^^+†^	Brazil	2005	1	3	22 (75%)	0	3500
Smith-Pardo & Gonzalez^91^^+†^	Colombia	1997	4	48	300 (46.2%)	0	nr
Parra-H & Nates-Parra^92^^+†^	Colombia	2003	1	26	21 (100%)	0	nr
Poveda *et al*.^93^^+†^	Colombia	2006–2007	2	34	4 (0%)	468	23
Tylianakis *et al*.^94^^+†^	Ecuador	2003–2004	1	48	16 (0%)	16	71
Vergara & Badano^64^^+†^	Mexico	2004	1	16	7 (71.43%)	8	nr
Fierro *et al*.^95^^†‡^	Mexico	2009–2010	1	3	4 (100%)	0	346.41
Rousseau *et al*.^96^^+†^	Nicaragua	2011	1	72	2 (100%)	81	30
	**Palearctic**		**64**	**2271**	**601**	**788**	
Verboven *et al*.^97^^†^	Belgium	2009	1	9	6 (66.67%)	0	11.34
Billeter *et al*.^98^^+†^, Diekötter *et al*.^99^^+†^ and Le Féon *et al*.^100^^+†^	Belgium, Czech Republic, Estonia, France, Germany, Netherlands, Switzerland	2001–2002	14	873	276 (98.46%)	7	nr
Kruess & Tscharntke^101^^+^	Germany	1996	2	34	17 (100%)	18	nr
Meyer *et al*.^102^^+†^	Germany	2000, 2005	2	30	14 (75%)	8	34.51
Diekötter *et al*.^103^^†^	Germany	2001	1	124	2 (100%)	0	353.55
Meyer *et al*.^104^,^105^^+†^	Germany	2004	1	32	109 (100%)	75	nr
Herrmann *et al*.^106^^†‡^	Germany	2005	2	26	1 (100%)	0	800
Holzschuh *et al*.^107^^+^	Germany	2007	2	134	3 (33.33%)	1	100
Weiner *et al*.^108^^+^	Germany	2007	1	29	59 (100%)	460	333
Nielsen *et al*.^109^^+†‡^	Greece	2004	4	32	1 (0%)	0	nr
Power & Stout^110^^+†^	Ireland	2009	1	20	9 (88.89%)	24	1200.24
Davis *et al*.^111^^†‡^	Ireland, United Kingdom	2005, 2007, 2008, 2009	1	12	1 (100%)	0	nr
Quaranta *et al*.^112^^+†^	Italy	2000	1	2	31 (100%)	0	200
Yoon *et al*.^113^	Korea, Republic of	2000–2012	1	215	6 (100%)	1	nr
Kohler *et al*.^114^^+†^	Netherlands	2004–2005	4	19	26 (95.48%)	56	1500
Goulson *et al*.^115^^†^	Poland	2006	1	32	22 (100%)	0	200
Mudri-Stojnic *et al*.^116^^+†‡^	Serbia	2011	1	16	55 (100%)	8	nr
Öckinger & Smith^117^	Sweden	2004	1	36	11 (100%)	64	800
Franzén & Nilsson^118^^+†^	Sweden	2005	1	16	83 (100%)	43	nr
Samnegård *et al*.^119^^+†^	Sweden	2009	1	9	31 (100%)	0	90
Oertli *et al*.^120^^+†^	Switzerland	2001–2002	1	7	237 (100%)	0	2000
Albrecht *et al*.^121^^+^	Switzerland	2003–2004	2	202	75 (100%)	0	nr
Farwig *et al*.^122^^+†^	Switzerland	2008	1	30	1 (0%)	0	nr
Schüepp *et al*.^123^^+†^	Switzerland	2008	1	30	11 (72.73%)	69	0.2
Darvill *et al*.^124^^†^	United Kingdom	2001	1	17	3 (66.67%)	0	100
Marshall *et al*.^125^^+†^	United Kingdom	2003	2	84	25 (100%)	0	nr
Hanley (2005, unpublished data)^†^	United Kingdom	2004–2005	1	6	11 (100%)	0	1000
Knight *et al*.^126^^†‡^	United Kingdom	2004	1	12	1 (100%)	0	3.16
Connop *et al*.^127^^†‡^	United Kingdom	2005	1	5	2 (100%)	0	nr
Goulson *et al*.^128^^†^	United Kingdom	2007	1	14	2 (100%)	0	200.25
Hanley *et al*.^129^^†^	United Kingdom	2007–2010	1	34	6 (100%)	0	200.04
Blake *et al*.^130^^†^	United Kingdom	2008–2010	2	6	8 (75%)	2	90
Redpath *et al*.^131^^†^	United Kingdom	2008	1	11	7 (85.71%)	0	nr
Bates *et al*.^132^^+†^	United Kingdom	2009–2010	1	24	58 (100%)	50	56.6
Osgathorpe *et al*.^133^^†^	United Kingdom	2009–2010	2	45	11 (90.91%)	1	nr
R. E. Fowler (PhD thesis, 2014)^+†^	United Kingdom	2011–2012	1	36	75 (100%)	0	nr
Hanley (unpublished data, 2011)^+†^	United Kingdom	2011	1	8	23 (82.61%)	110	nr

mMLE = largest Maximum Linear Extent (in meters) of any site in the source. MLE is the maximum distance between sampling points within a site, e.g. the length of a transect or the distance between pan traps. nr = not reported. Numbers of taxa are the numbers of unique taxa for which diversity measurements are given (so, if diversity measurements are available only for all bees combined, this would count as one taxon). The percentage of bee species with a known binomial name is also given (% binomial). Note that the figures here represent available data as curated by the PREDICTS team; these will not necessarily match figures in the original papers. ^+^Data were used in the presented analysis. ^†^Data will be incorporated into the PREDICTS database (which will be made openly available). ^‡^Data are available from the referenced paper. For all other datasets, please contact the corresponding author of that paper directly.
