# Supplementary material for: Predicting bee community responses to land-use changes: Effects of geographic and taxonomic biases
Source: Sci Rep. 2016 Aug 11;6:31153. doi: 10.1038/srep31153 (PMC4980681; doi:10.1038/srep31153)
Supplement: Supplementary Information [file srep31153-s1.pdf]

Supplementary Material: Predicting bee community responses to land-use changes: Effects of geographic and taxonomic biases

Adriana De Palma Stefan Abrahamczyk Marcelo A. Aizen
Matthias Albrecht Yves Basset Adam Bates Robin J. Blake
Céline Boutin Rob Bugter Stuart Connop Leopoldo Cruz-López
Saul A. Cunningham Ben Darvill Tim Diekötter Silvia Dorn
Nicola Downing Martin H. Entling Nina Farwig Antonio Felicioli
Steven J. Fonte Robert Fowler Markus Franzén Dave Goulson
Ingo Grass Mick E. Hanley Stephen D. Hendrix Farina Herrmann
Felix Herzog Andrea Holzschuh Birgit Jauker Michael Kessler
M.E. Knight Andreas Kruess Patrick Lavelle Violette Le Féon
Pia Lentini Louise A. Malone Jon Marshall
Eliana Martínez Pachón Quinn S. McFrederick Carolina L. Morales
Sonja Mudri-Stojnic Guiomar Nates Parra Sven G. Nilsson
Erik Öckinger Lynne Osgathorpe Alejandro Parra-H Carlos Peres
Anna S. Persson Theodora Petanidou Katja Poveda
Eileen F. Power Marino Quaranta Carolina Quintero
Romina Rader Miriam H. Richards T'ai Roulston
Laurent Rousseau Jonathan P. Sadler Ulrika Samnegård
Nancy A. Schellhorn Christof Schüepp Oliver Schweiger

Allan H. Smith-Pardo Ingolf Steffan-Dewenter Jane C. Stout
Rebecca K. Tonietto Teja Tscharntke Jason M. Tylianakis
Hans A.F. Verboven Carlos H. Vergara Jort Verhulst
Catrin Westphal Hyung Joo Yoon Andy Purvis

Supplementary Data S1. Relationship between sampled abundance and sampling effort

Generalized additive mixed models (Wood & Scheipl, 2014) were used to assess whether total abundance at a site was non-linearly related to sampling effort, for 18 studies where sampling effort varied among sites. Random effects included Source, Study and Block (see main text for details). A non-linear term for sampling effort was not supported although total abundance did significantly increase with sampling effort as expected (edf = 1, ref.df = 1, F = 7.111, $p < 0.01$).

Supplementary Table S1: Land-use class and intensity definitions as used in Hudson *et al.* (2014)

Level 1 Land Use	Predominant Land Use	Low Intensity	Medium Intensity	High Intensity
**No evidence of prior destruction of the vegetation**	**Primary forest**	Any disturbances identified are very minor (e.g., a trail or path) or very limited in the scope of their effect (e.g., hunting of a particular species of limited ecological importance).	One or more disturbances of moderate intensity (e.g., selective logging) or breadth of impact (e.g., bushmeat extraction), which are not severe enough to markedly change the nature of the ecosystem. Primary sites in suburban settings are at least medium intensity.	One or more disturbances that is severe enough to markedly change the nature of the ecosystem; this includes clear-felling of part of the site too recently for much recovery to have occurred. Primary sites in fully urban settings should be classed as high intensity.
	**Primary Non-Forest**	As above	As above	As above
**Recovering after destruction of the vegetation**	**Mature Secondary Vegetation**	As for Primary Vegetation-low intensity	As for Primary Vegetation-medium intensity	As for Primary Vegetation-high intensity
	**Intermediate Secondary Vegetation**	As for Primary Vegetation-low intensity	As for Primary Vegetation-medium intensity	As for Primary Vegetation-high intensity
	**Young Secondary Vegetation**	As for Primary Vegetation-low intensity	As for Primary Vegetation-medium intensity	As for Primary Vegetation-high intensity
	**Secondary Vegetation (indeterminate age)**	As for Primary Vegetation-low intensity	As for Primary Vegetation-medium intensity	As for Primary Vegetation-high intensity

**Human use (agricultural)**	**Plantation forest**	Extensively managed or mixed timber, fruit/coffee, oil-palm or rubber plantations in which native understorey and/or other native tree species are tolerated, which are not treated with pesticide or fertiliser, and which have not been recently (< 20 years) clear-felled.	Monoculture fruit/coffee/rubber plantations with limited pesticide input, or mixed species plantations with significant inputs. Monoculture timber plantations of mixed age with no recent (< 20 years) clear-felling. Monoculture oil-palm plantations with no recent (< 20 years) clear-felling.	Monoculture fruit/coffee/rubber plantations with significant pesticide input.Monoculture timber plantations with similarly aged trees or timber/oil-palm plantations with extensive recent (< 20 years) clear-felling.
	**Cropland**	Low-intensity farms, typically with small fields, mixed crops, crop rotation, little or no inorganic fertiliser use, little or no pesticide use, little or no ploughing, little or no irrigation, little or no mechanisation.	Medium intensity farming, typically showing some but not many of the following: large fields, annual ploughing, inorganic fertiliser application, pesticide application, irrigation, no crop rotation, mechanisation, monoculture crop. Organic farms in developed countries often fall within this category, as may high-intensity farming in developing countries.	High-intensity monoculture farming, typically showing many of the following features: large fields, annual ploughing, inorganic fertiliser application, pesticide application, irrigation, mechanisation, no crop rotation.
	**Pasture**	Pasture with minimal input of fertiliser and pesticide, and with low stock density (*not* high enough to cause significant disturbance or to stop regeneration of vegetation).	Pasture either with significant input of fertiliser or pesticide, or with high stock density (high enough to cause significant disturbance or to stop regeneration of vegetation).	Pasture with significant input of fertiliser or pesticide, *and* with high stock density (high enough to cause significant disturbance or to stop regeneration of vegetation).
**Human use (urban)**	**Urban**	Extensive managed green spaces; villages.	Suburban (e.g. gardens), or small managed or unmanaged green spaces in cities.	Fully urban with no significant green spaces.

Supplementary Data S2. List of species (with known binomial name) included in the dataset

Agapostemon melliventris

Agapostemon radiatus

Agapostemon semimelleus

Agapostemon texanus

Agapostemon virescens

Agapostemonoides hurdi

Aglaoapis tridentata

Allodape interrupta

Allodape mea

Allodapula variegata

Amegilla albigena

Amegilla asserta

Amegilla chlorocyanea

Amegilla pulchra

Amegilla quadrifasciata

Amphylaeus nubilosellus

Ancyloscelis apiformis

Andrena afrensis

Andrena agilissima

Andrena albofasciata

Andrena alfkenella

Andrena angustior

Andrena anthrisci

Andrena arabis

Andrena barbareae

Andrena barbilabris

Andrena bicolor

Andrena brevipalpis

Andrena bucephala

Andrena carantonica

Andrena carlini

Andrena chrysopus

Andrena chrysosceles

Andrena cineraria

Andrena cinerea

Andrena clarkella

Andrena coitana

Andrena combinata

Andrena commoda

Andrena congruens

Andrena cressonii

Andrena curvungula

Andrena decipiens

Andrena denticulata

Andrena distinguenda

Andrena dorsata

Andrena dunningi

Andrena enslinella

Andrena erigeniae

Andrena erythronii

Andrena falsifica

Andrena flavipes

Andrena florea

Andrena floricola

Andrena florivaga

Andrena forbesii

Andrena fucata

Andrena fulva

Andrena fulvago

Andrena fulvata

Andrena fulvida

Andrena fuscipes

Andrena gravida

Andrena haemorrhoa

Andrena hattorfiana

Andrena helianthiformis

Andrena helvola

Andrena hippotes

Andrena humilis

Andrena illinoiensis

Andrena intermedia

Andrena labialis

Andrena labiata

Andrena lagopus

Andrena lapponica

Andrena lathyri

Andrena mandibularis

Andrena minutula

Andrena minutuloides

Andrena miserabilis

Andrena mitis

Andrena nasonii

Andrena nasuta

Andrena nigrihirta

Andrena nigroaenea

Andrena nigroolivacea

Andrena nitida

Andrena nitidiuscula

Andrena niveata

Andrena ocreata

Andrena ovatula

Andrena pandellei

Andrena pilipes

Andrena polita

Andrena praecox

Andrena probata

Andrena propinqua

Andrena proxima

Andrena quintilis

Andrena ranunculorum

Andrena rosae

Andrena rudbeckiae

Andrena rufa

Andrena ruficrus

Andrena rufizona

Andrena sabulosa

Andrena saundersella

Andrena semilaevis

Andrena similis

Andrena spinigera

Andrena strohmella

Andrena subopaca

Andrena synadelpha

Andrena tarsata

Andrena thaspii

Andrena thoracica

Andrena tibialis

Andrena ungeri

Andrena vaga

Andrena varians

Andrena ventralis

Andrena viridescens

Andrena vulpecula

Andrena wheeleri

Andrena wilkella

Anthidiellum notatum

Anthidiellum strigatum

Anthidium byssinum

Anthidium caturigense

Anthidium laterale

Anthidium manicatum

Anthidium montanum

Anthidium oblongatum

Anthidium punctatum

Anthidium scapulare

Anthidium septemdentatum

Anthidium strigatum

Anthodioctes calcaratum

Anthodioctes mapirense

Anthophora aestivalis

Anthophora atroalba

Anthophora balneorum

Anthophora crassipes

Anthophora dispar

Anthophora furcata

Anthophora plumipes

Anthophora quadrimaculata

Anthophora retusa

Anthophora terminalis

Anthophora walshii

Apis mellifera

Augochlora cordicefloris

Augochlora nigrocyanea

Augochlora nominata

Augochlora pura

Augochlora repandirostris

Augochlora sidaefaline

Augochlorella aurata

Augochlorella edentata

Augochlorella striata

Augochloropsis auriventris

Augochloropsis callichroa

Augochloropsis metallica

Augochloropsis vesta

Bombus argillaceus

Bombus atratus

Bombus auricomus

Bombus barbutellus

Bombus bimaculatus

Bombus bohemicus

Bombus campestris

Bombus citrinus

Bombus cryptarum

Bombus dahlbomii

Bombus distinguendus

Bombus fervidus

Bombus gerstaeckeri

Bombus griseocollis

Bombus hortorum

Bombus humilis

Bombus hypnorum

Bombus impatiens

Bombus jonellus

Bombus lapidarius

Bombus lucorum

Bombus magnus

Bombus mesomelas

Bombus monticola

Bombus muscorum

Bombus norvegicus

Bombus pascuorum

Bombus pensylvanicus

Bombus pomorum

Bombus pratensis

Bombus pratorum

Bombus quadricolor

Bombus rapunculi

Bombus ruderarius

Bombus ruderatus

Bombus rufocinctus

Bombus rupestris

Bombus schrencki

Bombus semenoviellus

Bombus sichelii

Bombus soroeensis

Bombus subterraneus

Bombus sylvarum

Bombus sylvestris

Bombus ternarius

Bombus terrestris

Bombus vagans

Bombus vestalis

Bombus veteranus

Bombus wurflenii

Braunsapis calidula

Braunsapis diminuta

Braunsapis facialis

Braunsapis luapulana

Braunsapis simillima

Cadeguala albopilosa

Caenaugochlora perpectinata

Calliopsis andreniformis

Callohesma calliopsiformis

Callohesma ornatula

Callohesma pedalis

Camptopoeum frontale

Centris ferruginea

Centris flavifrons

Centris lubrosa

Centris obscurior

Centris varia

Cephalotrigona zexmeniae

Ceratina australensis

Ceratina calcarata

Ceratina chalybea

Ceratina chloris

Ceratina cucurbitina

Ceratina cyanea

Ceratina dupla

Ceratina eximia

Ceratina nigrolabiata

Ceratina placida

Ceratina regalis

Ceratina strenua

Ceratina viridicincta

Ceratina viridis

Ceratina zeteki

Ceylalictus perditellus

Chalepogenus caeruleus

Chalicodoma florisomne

Chelostoma campanularum

Chelostoma distinctum

Chelostoma florisomne

Chelostoma grande

Chelostoma rapunculi

Chlerogella elongaticeps

Coelioxys afra

Coelioxys banksi

Coelioxys conoidea

Coelioxys inermis

Coelioxys octodentata

Coelioxys rufescens

Coelioxys rufitarsis

Coelioxys simillima

Colletes americanus

Colletes compactus

Colletes cunicularius

Colletes daviesanus

Colletes floralis

Colletes fodiens

Colletes howardi

Colletes hyalinus

Colletes inflatus

Colletes latitarsis

Colletes marginatus

Colletes nigricans

Colletes robertsonii

Colletes seminitidus

Colletes sierrensis

Colletes simulans

Colletes succinctus

Colletes susannae

Corynura prothysteres

Dasypoda altercator

Dasypoda argentata

Dasypoda plumipes

Diphaglossa gayi

Dufourea dentiventris

Dufourea inermis

Dufourea minuta

Dufourea monardae

Epicharis albofasciata

Epicharis maculata

Epicharis monozona

Epicharis rustica

Eucera clypeata

Eucera eucnemidea

Eucera hamata

Eucera interrupta

Eucera longicornis

Eucera nigrescens

Eufriesea brasilianorum

Eufriesea concava

Eufriesea lucifera

Eufriesea macroglossa

Eufriesea mussitans

Eufriesea ornata

Eufriesea pulchra

Eufriesea purpurata

Eufriesea surinamensis

Euglossa allagticta

Euglossa analis

Euglossa bursigera

Euglossa chalybeata

Euglossa championi

Euglossa cognata

Euglossa cordata

Euglossa crassipunctata

Euglossa cyanura

Euglossa cybelia

Euglossa deceptrix

Euglossa dressleri

Euglossa fimbriata

Euglossa flammea

Euglossa hansoni

Euglossa hemichlora

Euglossa heterosticta

Euglossa ignita

Euglossa imperialis

Euglossa intersecta

Euglossa magnipes

Euglossa mixta

Euglossa modestior

Euglossa nigropilosa

Euglossa orellana

Euglossa parvula

Euglossa pleosticta

Euglossa sapphirina

Euglossa securigera

Euglossa singularis

Euglossa towasendi

Euglossa townsendi

Euglossa tridentata

Euglossa truncata

Euglossa variabilis

Euglossa viridis

Euglossa viridissima

Euhesma neglectula

Eulaema bombiformis

Eulaema cingulata

Eulaema meriana

Eulaema nigrifacies

Eulaema nigrita

Eulaema polychroma

Eulaema seabrai

Eulaema speciosa

Euryglossa adelaidae

Euryglossa depressa

Euryglossa edwardsii

Evylaeus calceatus

Evylaeus leucopus

Evylaeus morio

Evylaeus rufitarsis

Exaerete frontalis

Exaerete smaragdina

Exomalopsis artifex

Exoneurella setosa

Frieseomelitta nigra

Habralictus xanthinus

Halictus compressus

Halictus confusus

Halictus gemmeus

Halictus kessleri

Halictus langobardicus

Halictus leucaheneus

Halictus leucopus

Halictus ligatus

Halictus maculatus

Halictus parallelus

Halictus patellatus

Halictus pollinosus

Halictus quadricinctus

Halictus rubicundus

Halictus scabiosae

Halictus seladonius

Halictus semitectus

Halictus sexcinctus

Halictus simplex

Halictus smaragdulus

Halictus subauratus

Halictus tumulorum

Heriades carinatus

Heriades crenulatus

Heriades leavitti

Heriades truncorum

Heriades variolosa

Heriades variolosus

Honey bees

Hoplitis claviventris

Hoplitis lepeletieri

Hoplitis leucomelana

Hoplitis pilosifrons

Hoplitis producta

Hoplitis spoliata

Hyanth 0

Hylaeus affinis

Hylaeus albocuneatus

Hylaeus albonitens

Hylaeus amiculinus

Hylaeus angustatus

Hylaeus annularis

Hylaeus annulatus

Hylaeus asperithorax

Hylaeus brevicornis

Hylaeus chlorosomus

Hylaeus clypearis

Hylaeus communis

Hylaeus confusus

Hylaeus cressoni

Hylaeus cyanurus

Hylaeus difformis

Hylaeus gibbus

Hylaeus gredleri

Hylaeus hyalinatus

Hylaeus illinoisensis

Hylaeus kahri

Hylaeus lateralis

Hylaeus leptocephalus

Hylaeus mesillae

Hylaeus modestus

Hylaeus nigritus

Hylaeus nubilosus

Hylaeus paulus

Hylaeus punctatus

Hylaeus punctulatissimus

Hylaeus relegatus

Hylaeus rinki

Hylaeus signatus

Hylaeus sinuatus

Hylaeus stevensi

Hylaeus styriacus

Hylaeus variegatus

Isepeolus viperinus

Lasioglossum admirandum

Lasioglossum aeratum

Lasioglossum albipenne

Lasioglossum albipes

Lasioglossum albocinctum

Lasioglossum anomalum

Lasioglossum aspratulum

Lasioglossum asteris

Lasioglossum atlanticum

Lasioglossum bicingulatum

Lasioglossum bidentatulum

Lasioglossum brazieri

Lasioglossum brevicorne

Lasioglossum breviventre

Lasioglossum brisbanense

Lasioglossum calceatum

Lasioglossum callidum

Lasioglossum cambagei

Lasioglossum cinctipes

Lasioglossum clelandi

Lasioglossum clypeare

Lasioglossum coeruleum

Lasioglossum cognatum

Lasioglossum convexiusculum

Lasioglossum coriaceum

Lasioglossum corvinum

Lasioglossum costulatum

Lasioglossum cressonii

Lasioglossum cupromicans

Lasioglossum dampieri

Lasioglossum disabanci

Lasioglossum discum

Lasioglossum divergens

Lasioglossum dreisbachi

Lasioglossum ebeneum

Lasioglossum euboeense

Lasioglossum expansifrons

Lasioglossum fattigi

Lasioglossum foxii

Lasioglossum fratellum

Lasioglossum fulvicorne

Lasioglossum glabriusculum

Lasioglossum griseolum

Lasioglossum gynochilum

Lasioglossum helichrysi

Lasioglossum hemichalceum

Lasioglossum hiltacum

Lasioglossum illinoense

Lasioglossum imitator

Lasioglossum interruptum

Lasioglossum laeve

Lasioglossum laevigatum

Lasioglossum laevissimum

Lasioglossum lanarium

Lasioglossum laticeps

Lasioglossum lativentre

Lasioglossum leucopus

Lasioglossum leucozonium

Lasioglossum lineare

Lasioglossum lineatulum

Lasioglossum lissonotum

Lasioglossum lucidulum

Lasioglossum majus

Lasioglossum malachurum

Lasioglossum marginatum

Lasioglossum michiganense

Lasioglossum minutissimum

Lasioglossum minutulum

Lasioglossum morio

Lasioglossum mundulum

Lasioglossum nigripes

Lasioglossum nigroviride

Lasioglossum nitidiusculum

Lasioglossum nitidulum

Lasioglossum pallens

Lasioglossum paradmirandum

Lasioglossum paraforbesii

Lasioglossum parvulum

Lasioglossum pauperatum

Lasioglossum pauxillum

Lasioglossum pectorale

Lasioglossum peraustrale

Lasioglossum perpunctatum

Lasioglossum pilosum

Lasioglossum politum

Lasioglossum pruinosum

Lasioglossum punctatissimum

Lasioglossum punctatum

Lasioglossum puncticolle

Lasioglossum pygmaeum

Lasioglossum quadrinotatum

Lasioglossum quadrisignatum

Lasioglossum rohweri

Lasioglossum rufitarse

Lasioglossum sabulosum

Lasioglossum sagax

Lasioglossum sculpturatum

Lasioglossum semilucens

Lasioglossum sexnotatum

Lasioglossum sexsetum

Lasioglossum sexstrigatum

Lasioglossum smeathmanellum

Lasioglossum sordidum

Lasioglossum speculatum

Lasioglossum sphecodoides

Lasioglossum sphecodopsis

Lasioglossum subfasciatum

Lasioglossum sulthicum

Lasioglossum tegulare

Lasioglossum tricinctum

Lasioglossum uncinatum

Lasioglossum urbanum

Lasioglossum versatum

Lasioglossum villosulum

Lasioglossum viridatum

Lasioglossum willsi

Lasioglossum xanthopus

Lasioglossum zephyrum

Lasioglossum zonulum

Lasioglossum zophops

Leioproctus amabilis

Leioproctus carinatus

Leioproctus fulvescens

Leioproctus pango

Lestrimelitta niitkib

Liotrigona bottegoi

Lipotriches australica

Lipotriches excellens

Lipotriches flavoviridis

Lipotriches halictella

Lipotriches moerens

Lipotriches muscosa

Lipotriches semipallida

Lithurgus cornutus

Macropis europaea

Macropis fulvipes

Manuelia gayi

Manuelia postica

Megachile albisecta

Megachile alpicola

Megachile analis

Megachile apicalis

Megachile apicata

Megachile atrella

Megachile brevis

Megachile callura

Megachile campanulae

Megachile canifrons

Megachile captionis

Megachile centuncularis

Megachile circumcincta

Megachile clara

Megachile crassipes

Megachile discolor

Megachile ericetorum

Megachile ferox

Megachile heriadiformis

Megachile inermis

Megachile latimanus

Megachile leachella

Megachile ligniseca

Megachile lucidiventris

Megachile macularis

Megachile melanopyga

Megachile mendica

Megachile montivaga

Megachile nigriventris

Megachile oblonga

Megachile parietina

Megachile pilidens

Megachile pugnata

Megachile pyrenaea

Megachile pyrenaica

Megachile relativa

Megachile rotundata

Megachile semiluctuosa

Megachile sequior

Megachile serricauda

Megachile texana

Megachile versicolor

Megachile willughbiella

Megachile zaptlana

Megalopta centralis

Melecta albifrons

Melecta luctuosa

Melipona beecheii

Melipona compressipes

Melipona fasciata

Melipona fuliginosa

Meliponula lendliana

Melissodes agilis

Melissodes apicata

Melissodes bimaculata

Melissodes comptoides

Melissodes denticulata

Melissodes desponsa

Melissodes druriella

Melissodes subillata

Melissodes trinodis

Melitta dimidiata

Melitta haemorrhoidalis

Melitta leporina

Melitta nigricans

Melitta tricincta

Melitturga clavicornis

Meroglossa torrida

Mesocheira bicolor

Nannotrigona mellaria

Nannotrigona perilampoides

Nannotrigona testaceicornis

Nesocolletes paahaumaa

Nomada alboguttata

Nomada armata

Nomada articulata

Nomada bethunei

Nomada bifasciata

Nomada castellana

Nomada conjungens

Nomada denticulata

Nomada emarginata

Nomada erigeronis

Nomada fabriciana

Nomada facilis

Nomada femoralis

Nomada ferruginata

Nomada flava

Nomada flavoguttata

Nomada flavopicta

Nomada fucata

Nomada fulvicornis

Nomada goodeniana

Nomada guttulata

Nomada hirtipes

Nomada integra

Nomada lathburiana

Nomada leucophthalma

Nomada marshamella

Nomada melathoracica

Nomada miniuscula

Nomada obtusifrons

Nomada panzeri

Nomada pygmaea

Nomada ruficornis

Nomada rufipes

Nomada sexfasciata

Nomada sheppardana

Nomada signata

Nomada stoeckherti

Nomada striata

Nomada succincta

Nomada zonata

Nomia maneei

Osiris barrocoloradensis

Osiris mourei

Osiris panamensis

Osmia adunca

Osmia albiventris

Osmia anceyi

Osmia andrenoides

Osmia anthocopoides

Osmia atriventris

Osmia aurulenta

Osmia bicolor

Osmia brevicornis

Osmia caerulescens

Osmia campanularum

Osmia conjuncta

Osmia cornigera

Osmia dalmatica

Osmia florisomne

Osmia florisomnis

Osmia gallarum

Osmia labialis

Osmia leaiana

Osmia leucomelana

Osmia lignaria

Osmia loti

Osmia mitis

Osmia mustelina

Osmia parietina

Osmia praestans

Osmia pumila

Osmia rapunculi

Osmia rufa

Osmia simillima

Osmia spinulosa

Osmia submicans

Osmia tergestensis

Osmia truncorum

Osmia uncinata

Osmia villosa

Osmia xanthomelana

Oxytrigona daemoniaca

Oxytrigona mellicolor

Pachyprosopis eucalypti

Pachyprosopis flavicauda

Pachyprosopis haematostoma

Pachyprosopis trichopoda

Panurgus banksianus

Panurgus calcaratus

Panurgus dentipes

Paratetrapedia calcarata

Paratrigona isopterophila

Partamona bilineata

Partamona cupira

Partamona testacea

Pasites maculatus

Patellapis stirlingi

Peponapis limitaris

Peponapis pruinosa

Pereirapis rhizophila

Pereirapis semiaurata

Plebeia franki

Plebeia frontalis

Plebeia minima

Protandrena andrenoides

Protandrena bancrofti

Pseudapis diversipes

Pseudaugochlora graminea

Pseudopanurgus albitarsis

Rhinetula dentricus

Rhodanthidium septemdentatum

Rophites algirus

Rophites canus

Rophites quinquespinosus

Ruizantheda mutabilis

Ruizantheda proxima

Scaptotrigona mexicana

Scaptotrigona pectoralis

Scaptotrigona xanthotricha

Sphecodes albilabris

Sphecodes alternatus

Sphecodes banksii

Sphecodes crassus

Sphecodes dichrous

Sphecodes ephippius

Sphecodes ferruginatus

Sphecodes geoffrellus

Sphecodes gibbus

Sphecodes heraclei

Sphecodes hyalinatus

Sphecodes miniatus

Sphecodes monilicornis

Sphecodes niger

Sphecodes pellucidus

Sphecodes profugus

Sphecodes puncticeps

Sphecodes ranunculi

Sphecodes reticulatus

Sphecodes rufiventris

Sphecodes scabricollis

Sphecodes spinulosus

Sphecodes unknown

Stelis lateralis

Stelis punctulatissima

Stelis signata

Svastra obliqua

Svastrides melanura

Synhalonia hamata

Tetragonula carbonaria

Tetralonia macroglossa

Tetraloniella alticincta

Tetraloniella lyncea

Tetraloniella nana

Tetraloniella scabiosae

Thygater crawfordi

Thyreus waroonensis

Trachusa byssina

Triepeolus helianthi

Trigona amathea

Trigona angostula

Trigona corvina

Trigona dorsalis

Trigona fulviventris

Trigona fuscipennis

Trigona leucogastra

Trigona muzoensis

Trigona nigerrima

Trigona nigra

Trigona perangulata

Trigona recursa

Trigona subgrisea

Trigonisca buyssoni

Trigonisca schulthessi

Xylocopa imitator

Xylocopa lachnea

Xylocopa muscaria

Xylocopa transitoria

Xylocopa valga

Xylocopa violacea

Xylocopa virginica

Supplementary Methods. Decomposition of Mean Squared Error into Bias and Variance

Following Sheiner & Beal (1981), we decompose Mean Squared Error into $bias^2$ and $variance$ in the following way across all cross validation sets.

Prediction errors (pe) are defined as:

$pe = p_i - y_i$

where p is the prediction and y is the observation for the ith value.

Mean prediction errors (me) are a measure of $bias$:

$bias = mean(pe)$

Finally, the variance of the prediction error can be defined as the mean squared deviation of prediction errors from their mean:

$variance = (pe_i - me)^2$

Supplementary Table S2: Decomposition of Mean Squared Error (MSE) into $Bias^2$ and Variance (Sheiner & Beal, 1981). The square root of MSE is also given. Note that the bees dataset refers to models where all bees were assessed; the bombus dataset refers to models where bumblebees were compared with all other bees.

Dataset	Response Variable	Model	$MSE^{0.5}$ (± standard error)	$Bias^2$	Variance
bees	NULL	log(total abundance+1)	1.88(±0.0167)	0.43000	3.100
bees	LUI	log(total abundance+1)	2.08(±0.02)	0.80000	3.500
bees	Subregion	log(total abundance+1)	1.78(±0.0199)	0.22000	3.000
bees	LUI+Subregion	log(total abundance+1)	1.93(±0.0232)	0.47000	3.300
bees	LUI*Subregion	log(total abundance+1)	1.74(±0.0292)	0.30000	2.800
bees	NULL	Simpson's Diversity (D)	0.312(±0.0034)	0.00130	0.096
bees	LUI	Simpson's Diversity (D)	0.316(±0.00376)	0.00250	0.098
bees	Subregion	Simpson's Diversity (D)	0.3(±0.00322)	0.00021	0.090
bees	LUI+Subregion	Simpson's Diversity (D)	0.303(±0.00374)	0.00030	0.092
bees	LUI*Subregion	Simpson's Diversity (D)	0.297(±0.00315)	0.00140	0.087
bees	NULL	Species Richness	12.6(±1.04)	12.00000	160.000
bees	LUI	Species Richness	12.6(±1.04)	12.00000	160.000
bees	Subregion	Species Richness	12.4(±1.04)	8.80000	160.000
bees	LUI+Subregion	Species Richness	12.4(±1.04)	8.30000	160.000
bees	LUI*Subregion	Species Richness	12.2(±1.05)	11.00000	150.000
bombus	LUI*Subregion	log(total abundance+1)	1.84(±0.0296)	0.77000	2.600
bombus	LUI*Genus	log(total abundance+1)	2.13(±0.0288)	1.60000	2.900
bombus	LUI*Subregion*Genus	log(total abundance+1)	1.74(±0.0291)	0.77000	2.200
bombus	LUI*Subregion	Simpson's Diversity (D)	0.302(±0.00328)	0.00180	0.089
bombus	LUI*Genus	Simpson's Diversity (D)	0.306(±0.00221)	0.00260	0.091
bombus	LUI*Subregion*Genus	Simpson's Diversity (D)	0.285(±0.00311)	0.00260	0.079
bombus	LUI*Subregion	Species Richness	9.44(±0.677)	3.90000	89.000
bombus	LUI*Genus	Species Richness	9.49(±0.684)	1.40000	93.000
bombus	LUI*Subregion*Genus	Species Richness	7.63(±0.563)	2.00000	59.000

Supplementary Table S3: Spatial autocorrelation in residuals for minimum adequate model, conducted on each study in turn. Note that this table only includes studies where Moran's I returned a result; some tests failed because of too few datapoints, because the study either had no or too few neighbouring sites within 10km of one another, or because the test assumptions were not met.

Minimum adequate model	Number of studies showing evidence of autocorrelation	Test of equal proportions
Total abundance, bees	2	$\chi = 0.18, p = 0.55$
Simpson's diversity, bees	0	$\chi = 1.46, p = 0.89$
Species richness, bees	6	$\chi = 0.24, p = 0.31$
Total abundance, bombus	4	$\chi = 0.097, p = 0.38$
Simpson's diversity, bombus	3	$\chi = 0.00, p = 0.50$
Species richness, bombus	4	$\chi = 0.08, p = 0.39$

References

Hudson, L. N. *et al.* The PREDICTS database: a global database of how local terrestrial biodiversity responds to human impacts. *Ecology and Evolution* **4**, 4701–4735 (2014).

Sheiner, L. B. & Beal, S. L. Some suggestions for measuring predictive performance. *Journal of Pharmacokinetics and Biopharmaceutics* **9**, 503–160 (1981).

Wood, S. & Scheipl, F. gamm4: Generalized additive mixed models using mgcv and lme4 (2014).
